# Improving forensic care: Embracing recovery and rehabilitation in a Pre-discharge Unit

**DOI:** 10.1192/j.eurpsy.2026.11277

**Published:** 2026-07-31

**Authors:** S. M. Toparlak, H. Lawrence, F. Saliki

**Affiliations:** Oxford Health NHS Foundation Trust, Oxford, United Kingdom

## Abstract

**Introduction:**

Lambourn House is a 15-bedded mixed open forensic pre-discharge ward within the Low Secure Stream of Thames Valley Forensic Mental Health Services (TVFMH). We encourage patients to help meet their individual goals with the help of regular one to one sessions with the team, attendance at residents’ meetings and the Moving-on group. We support smooth transitions into the community and aim to reduce the overall length of stay in higher levels of security.

O’Connor et al. (2021) explored the experiences of eight forensic residents of a forensic step-down rehabilitation unit and emphasised that being in a less restrictive environment enabled greater goal-setting, increased independence and residents particularly valued input from a range of different professions in the MDT, which supported preparing them for the ‘outside world’. In align with this Best et al. (2017) outlined the importance of constructing a new recovery-based identity to contrast with that of the offending/substance-using identity. In addition, Figure 1 below represents the themes collated from service user perspectives in a study exploring transitions across the Thames Valley forensic mental health service (Boardman, Shanahan & Winder, 2019).

**Objectives:**

Disentangle complexities in the referral process Practical implementation of recovery-orientated practice and relational security Improve physical environment Celebrating and disseminating success Increasing bed occupancy Long term benefits such as biopsychosocial improvement

**Methods:**

Peer support workers: a role model and an expert by experience Role of MDT i.e. occupational therapist, social worker, psychologist, medical doctor Redefining and making the service & referral pathway known to others Reviewing bed occupancy Speaking to staff and service users about their experience and how to improve services. (Figure 1)

**Results:**

The ward philosophy & patient leaflet reviewed and updated As part of the new referrals process; patients are required to identify the objectives they expect to work towards, and these are reviewed 6 monthly in the form of a coproduced Personal Development Review (PDR) Therapeutic Activities Committee (TAC) Promoted co-production and patient involvement through therapeutic activity Risk formulation sessions for in-depth knowledge and understanding of the patient among clinicians Monthly teaching sessions led by MDT

**Image 1:**
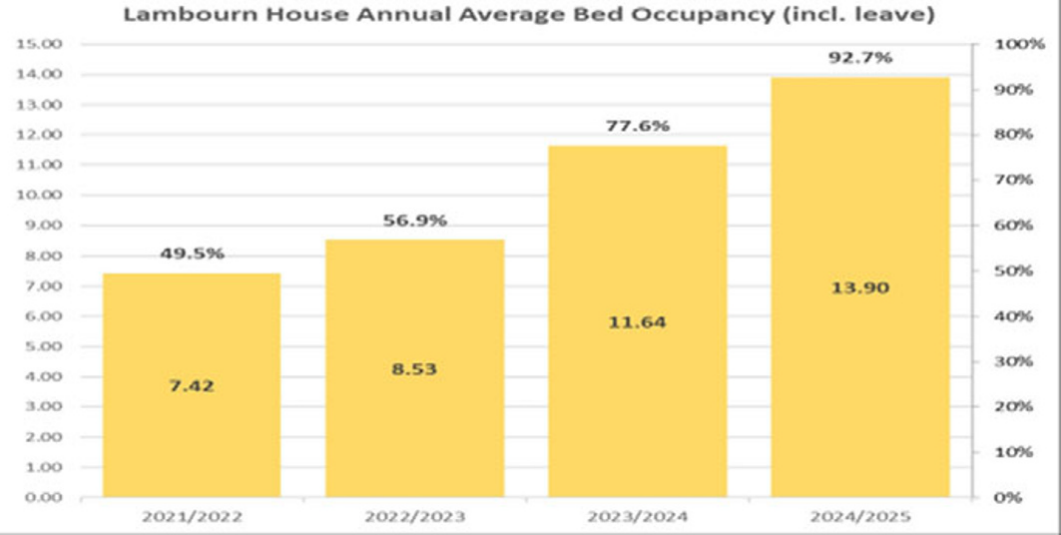

**Image 2:**
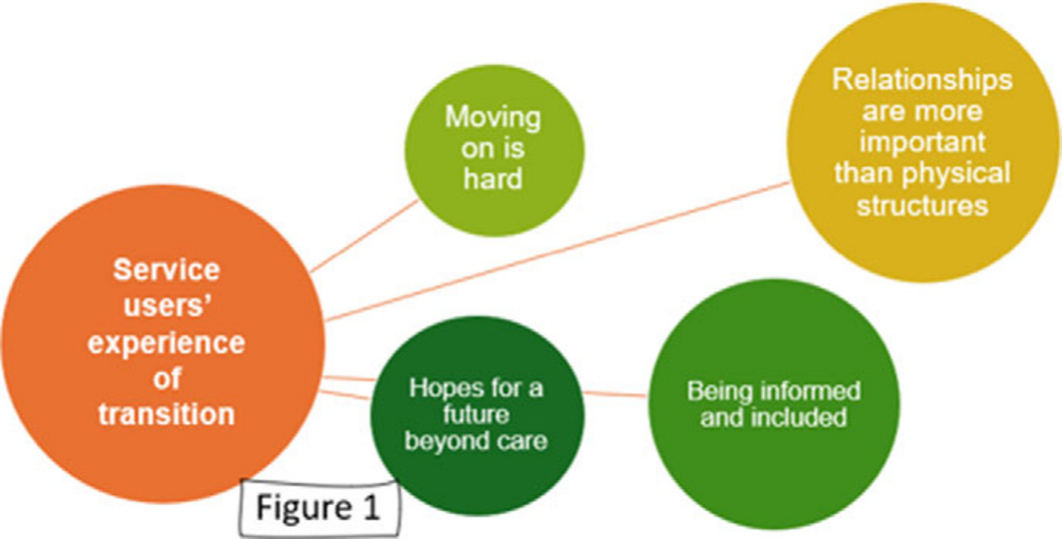

**Conclusions:**

There are currently **only three** forensic pre-discharge units in the UK, located **in Oxford, London, and Manchester.**

Raising awareness of the role and value of forensic pre-discharge units is essential so that similar services can be developed and replicated across the country.

**Disclosure of Interest:**

None Declared

